# Research on the construction of an adaptive mindfulness-based cognitive therapy system based on large language models and its efficacy in addressing college students’ anxiety

**DOI:** 10.3389/fpsyg.2026.1800278

**Published:** 2026-10-16

**Authors:** Yunlong Jia

**Affiliations:** School of Physical Education, Wuhan Sports University, Wuhan, China

**Keywords:** adaptive system, college students’ anxiety, large language model, mindfulness-based cognitive therapy, psychological intervention

## Abstract

To handle the problem of resource scarcity and lack of personalization and adaptability in the traditional approach to mindfulness-based cognitive therapy (MBCT) in dealing with college students with anxiety disorders, this study proposes the use of the natural language interface and adaptability properties of large language models (LLMs) to come up with an intelligent college-level MBCT intervention system. To begin with, the system functional requirements and the logic of the system intervention were explained in line with the fundamental ideas of MBCT and the cognitive features of students with anxiety. Second, it was created through a four-stage adaptive architecture that includes perception analysis, intervention, and feedback, providing a dynamic process for user status identification, personal generation of intervention plans, and real-time optimization of the intervention process. Finally, there was a randomized controlled trial whose subjects were 120 college students with anxiety who were separated into an experimental group and a control group. The effectiveness of the intervention was assessed using tools such as the Self-Rating Anxiety Scale (SAS), the Mindful Attention Awareness Scale (MAAS), and the Automatic Thoughts Questionnaire (ATQ). The findings indicated that the SAS score of the experimental group (38.26 ± 5.12) was significantly less than that of the control group (45.89 ± 6.34), the MAAS score (68.73 ± 7.25) was significantly higher than that of the control group (56.41 ± 8.13); and the ATQ score (42.15 ± 6.08) was significantly less than that of the control group. The study reveals that an adaptive MBCT system using large language models may be effective in reducing anxiety symptoms among college students, improving mindfulness, and enhancing cognitive processes, offering a new, readily available strategy for addressing anxiety in college students.

## Introduction

### Research background

The problem of anxiety as a characteristic of university students has taken center stage in recent years, introducing an essential burden and serving as a key parameter influencing the physical and mental health of students as well as their academic progression (Segal et al., 2002). As stated in the 2024 Report on the Development of Mental Health in University students, the percentage of university students in our country who reported having experienced anxiety reached 32.8%, including 21.3% reporting mild levels of anxiety and 11.5% reporting moderate or higher levels of anxiety. The consequences of anxiety on a university student do not solely incorporate inability to concentrate properly and sleeping disturbances. Still, they can also result in harmful psychological crises like depression and mental distress. In addition, there is a need for effective and active scientific intervention (Kabat-Zinn, 2003).

Mindfulness-based cognitive therapy (MBCT), as a technology of psychological intervention, involving the combination of daily mindfulness meditation and cognitive behavioral therapy, has proven to have enormous potential in the management of anxiety by increasing awareness and acceptance of the current situation, reducing negative patterns in thinking, and demonstrating high levels of efficacy in safeguarding against anxiety (Hofmann et al., 2010). Nevertheless, there are numerous limitations to the traditional MBCT interventions: (1) professional intervention resources are scarce; (2) the number of qualified mental health teachers and students of the university: the proportion of qualified professionals to students is around 1:4500, which can negatively affect the intervention requirements on a mass scale; (3) the current intervention program is highly standardized, and in-person intervention may be limited in time and space, which means that the intervention compliance rate is low (Brown and Ryan, 2003).

The technical facilitation of establishing psychological intervention models has been involved with the rapid creation of large language models (LLMs) like GPT-4 and Wen Xin Yiyan (Zung, 1971). They possess fundamental skills in natural language cognition, the production of individualized content, and interactive prompts that can discern the logic of consultations for professional advisors and match the intelligence and productivity of operations (Spielberger et al., 1983). The combination of LLM and MBCT has the potential to develop a dynamic intervention system that dismantles the solidarity of traditional interventions and provides a fresh solution for anxiety interventions in the lives of university students. In this respect, this study is dedicated to the advanced practice of system-building—achieving effectiveness and examining the procedure for applying the aforementioned LLM concept in MBCT interventions, providing theoretical and practical support for the creation of mental health service systems for university students (Hollon and Kendall, 1980; Hoge et al., 2013; Khosrowabadi et al., 2023; Radford et al., 2018).

### Research objectives and significance

The key inquiries of this research are: (1) Construction of a flexible MBCT system based on LLMs, creation of the system architecture, prime modules, areas of interaction, and realization of personal generation and dynamic enhancement of the intervention plan; (2) Applying the system to anxiety interventions among college students and evaluating its effectiveness by comparing improvements in anxiety symptoms, mindfulness awareness, and cognitive patterns; (3) Identifying key technical insights and intervention principles for integrating MBCT with LLMs to inform the development of intelligent psychological intervention systems (Brown et al., 2020; Li et al., 2024; Zhang et al., 2022).

Theoretical importance: There are no studies on intensive MBCT interventions; this work broadens the field of application of LLMs to mental health, and the combination of technologies and theory to create a psychological intervention digital transformation underlies theoretical arguments offered in the study. Practical importance: The new system can decrease professional obstacles to anxiety management, increase accessibility and ease, provide useful and incorporated tools to mental health teaching in the college, and help improve the psychological cognition of the university student (Karyotaki et al., 2017; Firth et al., 2017; Linardon et al., 2019; Kessler et al., 2007).

### Current research status at home and abroad

The development of MBCT in other countries has been rather slow, and various intervention systems have been offered. To illustrate, the Mindfulness Coach system combines cultural reflection and psychological assessment tools to support the attainment of a specific intervention management approach, yet it lacks the capability to make personal adjustments. However, in recent years, research has begun to emerge with the advent of artificial intelligence. An example is the MBCT intervention system developed by Khosrowabadi et al. (2023), which transforms the intervention plan based on users’ feedback on the reflection and enhances the reflection plan by analyzing the reflection data on the receiving side. Still, the methods of interaction are rather rigid, and the algorithm depends on the data received. In terms of the use of LLMs, the psychological support robot created by OpenAI in collaboration with Stanford University can replicate the process of MBCT guidance through natural language interaction, yet professional intervention and adaptive changes are still to be improved (Chiesa and Serretti, 2011).

Studies conducted within the local setting have been more on the impact of traditional MBCT on anxiety in university students. To illustrate one such example, Zhang et al. (2022) stated that the anxiety of university students can be reduced substantially with involvement in MBCT programs. Nonetheless, studies concerning intelligent MBCT are still new. The current intelligent intervention systems are rather obedient to cognitive behavioral therapy and mere emotional advice, as on sites such as Xiaoxin Psychology and Yi Psychology, and are open to the development of a systematic intervention with the theory of MBCT in mind. The incorporation of LLMs into psychological interventions is at the exploration phase. As an example, Li et al. (2024) examined the usefulness of LLMs in psychological assessment and omitted the entire incorporation with MBCT interventions (Levin et al., 2017; Twomey et al., 2015; Richards and Richardson, 2012; Fulmer et al., 2018; Guntuku et al., 2019).

In short, even though the internal and international studies have laid the groundwork for integrating LLMs and MBCT, there are still blatant gaps: first, the current tools are interested in the isolated cycles involving the dynamic assessment-personal intervention-temporal relationships; second, the implementation of the tools is aimed at a university student population and has not delved into the core of the MBCT intervention logic; third, the formulation and validation of the tools is quite weak. The research will address the above gaps by constructing a state-of-the-art MBCT system and carrying out experimental research to bridge a research gap in related studies (Torous et al., 2018; Chandrashekar, 2018; Lattie et al., 2019).

## Core theoretical foundation

### Core theory of mindfulness-based cognitive therapy (MBCT)

In 1992, Segal, Williams, and Teasdale suggested MBCT. It relies on the fundamental principles of mindfulness training combined with cognitive behavioral therapy (CBT), with a primary focus on the rationale for the mindfulness-acceptance-cognitive restructuring intervention. According to the theory, anxiety is closely connected with the so-called ruminative focus on negative thoughts and automated cognitive arousal, meaning that people tend to respond to or even avoid unpleasant emotions and other related negative thoughts, which also makes the process of experiencing anxiety persistent. Nevertheless, the negative thinking cycle may be interrupted through mindfulness training by building the ability to be fully present, in the current moment, so that people do not judge their thoughts and emotions and instead restructure thinking patterns (Garrido et al., 2019).

Classical MBCT intervention entails 8 weeks of group training sessions, the main techniques applied included mindfulness of the body and breath, cognitive therapies such as thought labeling and acceptance and commitment training, and values clarification exercises. Out of the group of university students with anxiety, the MBCT interventions and mechanisms can be introduced based on the core mechanisms of academic stress, negative interpersonal conflict, and future avoidance, which should be improved with the help of specific training of what is known as the affective skills-awareness-acceptance-mindfulness training. This provides a theoretical rationale for the development of a methodological approach to intervention content (Miner et al., 2020).

### Technical features and application logic of large language models (LLMs)

Large language models are very successful at training and, when trained on large datasets, containing hundreds of people, gain profound insight and sophisticated skills in natural language. Some of their central technical characteristics are, first, contextual awareness through which emotional states and cognitive abilities can be tested, which entails user intervention by multiple levels of interaction leading to supportive data to support specialized interventions, that is, beyond the preset topic constraints; second, the ability to produce content, that is, able to create intervention topics based on MBCT theories to fulfill user needs, that is, beyond the constraining factors of predefined topics; and third, the engaging ability, that is, an ability to gain user trust and participation in interventions by simulating the communication patterns of professional consultants (Carron-Arthur et al., 2015).

As a manifestation of the logic of integrating LLMs and MBCT, the implementation of MBCT intervention processes as an alternative implementation process empowers the natural language interaction with LLMs through intervention indicators (such as creating checked content in higher-order thinking due to the impact of user anxiety triggers); allows advanced learning power through customization of intervention plans (such as analysis of user opinion texts to infer emotional changes); and the data processing power enables the unforeseen evaluation of intervention effects (such as analysis of user opinion texts to estimate emotional changes) (Wang et al., 2018). This enhances access to MBCT interventions, monitoring, and effectiveness.

### Cognitive characteristics and intervention needs of college students’ anxiety

The cognitive features of anxiety among college students have both group variations: first, the antecedent factors include a wide range of factors, including academic pressure (failing tests and essays and writing articles), interpersonal influence (disputes in the dormitory and social anxiety), career development (not knowing what to do in life, being forced to take college entrance examinations), and emotional issues, second, cognitive bias are also prominent, with a tendency toward negative thinking processes such as catastrophizing (such as one failed examination can destroy my life) and absolutist demands (such as everybody should agree with me), which means that the This results to definite directions of an efficient design of a MBCT system.

### Construction of an adaptive MBCT system based on large language models

#### System design principles

According to the demands of the MBCT theory, the peculiarities of the LLC technology, and the needs of the university student, the system is formed systematically, taking into account four fundamental principles: the principle of professionalism, according to which all the content of the intervention should be checked with reference to psychology experts, strictly following the intervention logic of MBCT, not recommending any unprofessional advice; the principle of adaptability, according to which the content, difficulty, and frequency of the intervention program may be dynamically adjusted based on the basic information of the user, the results of the anxiety analysis, and the feedback.

#### System architecture design

The system is based on the four-stage closed-loop round of perception analysis and intervention feedback, following the browser/service (B/S) model, and can be broadly separated into three levels: perception layer, service layer, and application layer. The levels vary relative to one another to highlight the adaptive functions of intervention. This particular arrangement is as follows:

1. *Perception layer*: This is the fundamental layer whose role is to gather user status information, both static and dynamic information. The basic user data (gender, major, grade) and the anxiety baseline test (calculated using standardized scales) are considered static data. The interaction texts in the intervention process (such as user descriptions of emotions), meditation training feedback (such as focus status and emotional changes), and the use of indirect physiological indicators (e.g., heart rate variability, which is estimated with the help of the phone sensors) are referred to as dynamic data. The layer of perception uses three modes for achieving real-time data collection, including natural language input, scale questionnaires, and sensor data interfaces.
2. *Service layer*: It is the heart and soul of the system and incorporates the LLM engine, MBCT intervention engine, and data processing engine. The LLM engine is the fine-tuned WenXinYiYan model that incorporates MBCT professional logic via prompt engineering to produce natural language understanding and suggestions to personalize content; the MBCT intervention engine maintains an 8-week standardized intervention process and a core technology library to offer support in theoretical development of the intervention plan; the data processing engine analyzes the data of the perception layer and produces the user anxiety assessment report and intervention adjustment guidance, and provides adaptive optimization of the intervention plan.
3. *Application layer*: It consists of user-facing functional modules, and the modules are five in number, namely, User Center, Anxiety Assessment, Adaptive Intervention, Progress Tracking, and Crisis Alert. These modules are interrelated and form a chain of functions within the intervention.

## Core function module development

### Anxiety assessment module

*Module function*: To obtain a comprehensive assessment and dynamic monitoring of users’ anxiety, which will be used to develop an agenda of intervention plans. The two assessment methods that have been subdivided in the module include, first, standardized scale assessment, which comprises the Self-Rating Anxiety Scale (SAS), the State–Trait Anxiety Inventory (STAI), and the Automatic Thoughts Questionnaire (ATQ). A user completes the baseline assessment each time they use the app, and the dynamic assessment every week during the intervention period. Second, the natural language assessment: in this instance, the LLM analyzes the feelings and thoughts in the user’s writing according to multi-turn communication (e.g., what has made you anxious in the past week?). The focus on the issues in these questions makes this the extra evaluation report made (asking): What are your views on these things?

*Technical implementation*: It is implemented using a scale-and-text data fusion analysis model. The LLM defines the textual components of emotions through emotion analysis algorithms (i.e., the Valence Aware Dictionary and sEntiment Reasoner [VADER] sentiment analysis tool) and converts them into scale scores and the outcomes of the assessment, that is, the degree of anxiety, core triggers, and types of cognitive bias.

### Adaptive intervention module

*Module function*: It is the most important module of the system, as it creates personalized MBCT intervention programs based on the assessment results and optimizes them in real time in response to user feedback. The intervention procedure is based on the 8-week MBCT program. It consists of four parts: “Basic Mindfulness Training Period (Weeks 1-2) and Cognitive Awareness Period (Weeks 3-4), Cognitive Restructuring Period (Weeks 5-6), and Consolidation and Application Period (Weeks 7-8). LLM creates the intervention content on a stage-by-stage basis.

*Core intervention design*:

1. Mindfulness guidance: The LLM creates personalized meditation instructions and guidance depending on the situation in which the user is (e.g., before bed and during class break) and their individual preferences (e.g., the style of guidance and playing music). As an illustration, with users who have academic anxiety, the advice includes the aspect of maintaining concentration on the present study task; whereas, with users who have social anxiety, the advice focuses on the aspect of body sensations. Instructions can also be given in both voice and text, leaving the user to decide the format they will use.
2. Cognitive defusion: The LLM applies MBCT techniques, including thought labeling and story-based metaphors based on the negative thoughts of the user (e.g., I will fail the exam). Thus, by putting questions such as the following: What does this thought resemble—a cloud or a rock? The user would be able to dissociate themselves from negative thoughts, minimizing cognitive rumination.
3. Cognitive restructuring: This focuses on the core cognitive biases of the user, and through Socratic-style questioning, the LLM facilitates cognitive realignment. In the case of users exhibiting catastrophic thinking, ask questions such as What is the evidence behind this thought? might be used. and “Are there other possible outcomes? assist them in having diversified thinking.
4. Practical activities: The module has exercises of mindfulness that are built into daily practice, that is, paying attention to the taste and feel of the food when eating, or practicing 3 min of breath awareness during a break in a lesson can be trailed with the help of a check-in feature.
5. Adaptive adjustment mechanism: The LLM examines the user’s responses to interventions (e.g., Was I focused during this meditation?). Dynamic assessment outcomes and results adjust the difficulty of the intervention content (e.g., raising the length of meditation sessions by 5-15-min steps), areas to focus on (e.g., changing the intervention core based on occurrences of anxiety triggers), and format (e.g., should the user report that voice guidance is easier to concentrate on, the system prioritizes voice guidance).

### Progress tracking and feedback module

*Function of the module*: To enable visualization of the intervention process and provide separate feedback, thereby enhancing user compliance. The module has two main functions. First, progress visualization presents users with their intervention adherence rates, trends in anxiety scores, and changes in mindfulness awareness through charts, enabling them to monitor their progress. Second, personalized feedback provides users with weekly intervention reports. The LLM analyzes individual progress, highlights improvements in anxiety symptoms and mindfulness, and offers personalized suggestions to support continued engagement with the intervention.

### Crisis warning module

*Module function*: Recognize the risk of extreme anxiety and provide psychological stability among the users. The module detects risks on two counts: first, a warning on the scale score; the user with a SAS score of 60 or above (severe anxiety) or a statement mentioning suicidal ideas; the warning therefore automatically occurs; and second, a warning on the text emotion; that is, the system monitors the intensity of negative emotions in the texts exchanged by the user in real-time, and the warning is brought up in the event of extremely negative emotions and persistence. Once a warning is sent, the system instantly provides psychological support resources (including means of appointment to university counseling centers or 24-h psychological assistance hotlines) and coordinates the information about the warning with the instructors in university psychology (with the user’s consent).

### System performance research design

#### Research hypotheses

Based on theoretical analysis, this study proposes three core hypotheses:

*Hypothesis H1*: After intervention with the LLM-based adaptive MBCT system, the anxiety levels (SAS, STAI scores) of college students in the experimental group will be significantly lower than those in the control group.

*Hypothesis H2*: After intervention with the LLM-based adaptive MBCT system, the mindfulness levels (MAAS scores) of college students in the experimental group will be significantly higher than those in the control group.

*Hypothesis H3*: After intervention with the LLM-based adaptive MBCT system, the negative cognitive levels (ATQ scores) of college students in the experimental group will be significantly lower than those in the control group, and the intervention compliance rate will be higher than that of the control group.

#### Research subject

The convenience sample was comprised of a study group of undergraduate students in a specific university of the cohort of the 2024 generation, whose needs had to be filtered by the following criteria:

##### Inclusion criteria

(1) Score in SAS 50 points or higher (moderate anxiety) and (2) oc II two-state institutional history of mentally ill (depression or schizophrenia); 33 not previously trained in MBCT or other anxiety treatment methods; 43 voluntary participants in this study and managed to sign informed consent.

##### Exclusion criteria

(1) Severe psychological crisis at the time of the intervention and requires a referral to professional treatment; (2) excessive rate of data completion (less than 60); and (3) data reporting is evidently falsified.

After the first screening, 120 eligible subjects were randomly assigned to the experimental and control groups using a random number table, with 60 subjects in each group. The experimental sample consisted of 28 men and 32 women; average age (20.32 = 1.25) years; majors: 22 humanities, 18 science, 20 engineering. The control group consisted of 26 men and 34 women; mean age (20.18 ± 1.31 years); majors: 20 humanities, 21 science, 19 engineering. There were no statistically significant differences between the two groups with regard to gender, age, major, or baseline SAS scores (*p* > 0.05), which presupposes comparability.

#### Research tools

1. Self-Rating Anxiety Scale (SAS): Developed by Zung in 1971, it consists of 20 items, scored on a scale of 1–4. A total score of ≥50 indicates a positive anxiety result, with higher scores reflecting greater anxiety levels. This scale has good reliability and validity among college students, and the Cronbach’s *α* coefficient in this study was 0.83.
2. Mindful Attention Awareness Scale (MAAS): Developed by Brown and Ryan in 2003, it consists of 15 items, scored on a scale of 1–6. Higher scores indicate higher levels of mindfulness. The Cronbach’s *α* coefficient in this study was 0.81.
3. Automatic Thoughts Questionnaire (ATQ): Developed by Hollon and Kendall in 1980, it consists of 30 items, scored on a scale of 1–5. Higher scores indicate more frequent negative automatic thoughts. The Cronbach’s *α* coefficient in this study was 0.85.
4. Intervention Compliance Questionnaire: A self-designed questionnaire including three dimensions: “intervention completion rate,” “intervention concentration,” and “intervention satisfaction,” with a total of 6 items scored on a scale of 1–5. A total score of ≥24 indicates high compliance. The Cronbach’s *α* coefficient in this study was 0.78.

#### Research process

The research time was 10 weeks, and the time was broken down as follows: preparation phase, intervention phase, and follow-up phase:

1. *Preparation phase (week 1)*: Both groups of participants were assessed on a baseline level, and the fundamental data were gathered based on the SAS, MAAS, and ATQ. The experimental group was trained on using the system, and system functions and intervention requirements were explained to them; the control group was trained on traditional MBCT interventions.
2. *Intervention phase (weeks 2–9)*: The two groups were provided with an 8-week MBCT intervention with 3 days per week, with each session taking 10–20 min. The experimental group followed self-directed interventions using adaptive MBCT systems designed for this study, which dynamically adjusted intervention content based on user feedback; the control group followed the conventional MBCT group intervention model delivered by a psychologist with an intermediate MBCT certificate. The groups had 15 participants each, and there was 1 in-person group session lasting 60 min in person and 2 weekly online self-practice sessions (the content was the same as that used in the experimental group, but the self-practices were standardized, with no personalization).
3. *Follow-up phase (week 10)*: The group took repeated SAS, MAAS, and ATQ examinations, as well as an intervention compliance questionnaire to give the group post-test data; 10 participants of the experimental group were also interviewed semi-structured to gain insight into their experience of using the system and feedback about the intervention.

## Data processing

The Statistical Package for the Social Sciences (SPSS version 26.0) was used to process the data. Data were then cleaned, first by removing invalid data (partial skills between questionnaire answers or intervention compliance below 60%). A second analysis used descriptive statistics to determine the demographics and scale scores for participants in both groups. Finally, the post-intervention scale scores of the two groups were compared using independent-sample *t*-tests, and the differences in intervention compliance between the two groups were compared using Chi-square tests at a significance level of 0.05.

## Research results

### Completion status of interventions for the two groups of subjects

Some participants dropped out of the experimental group (4 due to academic workload; intervention completion rate 93.331) and controls (9 due to scheduling conflict; 3 due to poor experience with the intervention; intervention completion rate 85.001). The final valid sample comprised 107 participants, with 56 in the experimental group and 51 in the control group.

### Comparison of scale scores before and after intervention in two groups of subjects

Prior to the intervention, the two groups did not differ in SAS, MAAS, and ATQ scores (*p* > 0.05), indicating that the two methods compared were comparable. After the intervention, the SAS and ATQ scores in the control group were significantly lower than those in the experimental group (*p* < 0.05), whereas the MAAS score was higher in the control group than in the experimental group (*p* < 0.05). Detailed data are shown in Table 1.

**Table 1:** Comparison of SAS, MAAS, and ATQ scores between the experimental and control groups before and after the intervention.

Scale	Group	Before intervention (*M* ± SD)	Post-intervention (*M* ± SD)	*t*-value before intervention	*t*-value after intervention
SAS	Experimental group	56.32 ± 5.41	38.26 ± 5.12	0.52	6.89
	Control group	55.89 ± 5.67	45.89 ± 6.34		
MAAS	Experimental group	50.12 ± 7.34	68.73 ± 7.25	0.38	8.23
	Control group	49.87 ± 7.51	56.41 ± 8.13		
ATQ	Experimental group	65.34 ± 7.89	42.15 ± 6.08	0.45	9.56
	Control group	64.98 ± 8.12	53.67 ± 7.42		

*M*, mean; SD, standard deviation; *p* < 0.01.

### Comparison of intervention compliance and satisfaction between the two groups of subjects

The intervention adherence rate in the experimental group was 86.67% (48/56), significantly higher than the 65.00% (33/51) observed in the control group (*χ*^2^ = 6.72, *p* < 0.01). In terms of intervention satisfaction scores, the experimental group had an average score of 28.32 ± 3.15, significantly higher than the control group’s 22.15 ± 4.21 (*t* = 7.89, *p* < 0.01).

Feedback from interviews with participants in the experimental group showed that 80% believed that the system’s “personalized guidance” and “convenience” were the main reasons for their high adherence, with comments such as, “The system adjusts the meditation content according to my mood that day, which is more considerate than fixed materials,” and “I can complete the intervention in 10 min between classes without having to go to an offline venue.” Additionally, 70% of the participants indicated that “natural language interaction makes me more willing to express my true emotions,” noting that the system’s human-like communication increased their trust in the intervention.

## Discussion

### Analysis of the intervention effectiveness of an adaptive MBCT system

The study findings show that the adaptive MBCT system based on LLC can significantly lower anxiety levels among college students, improve mindfulness skills, and reduce negative automatic thoughts, with effects stronger than those of conventional MBCT interventions, thereby supporting research hypotheses H1, H2, and H3. The root cause of this consequence is the high level of integration of the past theory of MBCT and the theory of the newest technology of LLM, which breaks the shortcomings of conventional approaches:

*(1) Personalized interventions increase adaptability*: The system is dynamic and captures anxiety triggers and cognitive features of users based on the context-sensitive features of LLM, creating specific intervention material. As an example, for users with academic anxiety, it emphasizes so-called task-oriented mindfulness training. In contrast, for users with social anxiety, it has an emphasis on emotion acceptance exercises. It is this very adaptation that makes the intervention more individualized and hence more effective. In contrast, the traditional MBCT employs standardized content, which limits its ability to accommodate individual differences and can offer a less gratifying user experience for some users.

*(2) The system enhances compliance*: The system promotes multi-device, on-demand interventions, and single sessions last no more than 10–20 min; the perfect time to fit the study profits of the university students. Natural language interaction mode mimics a conversation with a professional counselor, increasing users’ willingness to communicate. Some of these people, in interviews, stated that I do not have to set up offline appointments; I can open the system any time I am in a bad mood, and I say that chatting with the system is like chatting with a friend, making me more willing to use the system. This is in line with the intervention adherence results (experimental group 86.67% vs. control group 65.00%), which indicate that convenience and interactivity are critical elements in the maintenance of interventions.

*Closed-loop feedback mechanism in the optimization of the intervention process*: the system is controlled by closed-loop feedback with assessment intervention and feedback, adjusting the intervention plan in real time. For instance, when a user reports that it is difficult to focus in the process of meditating, the system will automatically reduce the time spent on meditation and give more guidance; when dynamic evaluation indicates slow progress of anxiety scores, cognitive restructuring training will be enhanced. This dynamic optimization will put the intervention in a healthy cycle of “adaptation-adjustment-readaptation,” making it more precise.

### Key technologies and theoretical insights in system construction

The key technical points in system construction are reflected in two aspects: first, the Prompt engineering design of LLMs, which guides the model to generate intervention content that meets professional requirements by incorporating MBCT terminology, intervention logic, and cognitive characteristics of college students into the prompt, thereby avoiding “generalized answers”; second, the implementation of adaptive algorithms, which, based on users’ assessment data and feedback, establishes a mapping relationship of “anxiety level—intervention content—adjustment strategy,” enabling the automated optimization of intervention plans. This provides a technical reference for the subsequent development of intelligent psychological intervention systems.

In terms of theoretical insights, this study validates the feasibility of “technology-enabled psychological intervention” and expands the application scenarios of MBCT. Traditional MBCT relies on face-to-face guidance from professionals. In contrast, this study achieves a “deprofessionalization threshold” of MBCT interventions through LLMs, making high-quality psychological intervention resources more accessible to college students and providing theoretical support for universal mental health services. Additionally, the study finds that college students have a relatively high acceptance of intelligent intervention tools, providing a population basis for the digital transformation of psychological interventions.

### Research limitations and the future prospects

The limitations of this study are as follows: first, the sample size is not very representative, as only undergraduates of one university were chosen, and it is hard to conclude about the results of a systematic intervention in another region and at another educational level; second, observation was based predominantly on self-reports and no objective data support (e.g., cortisol levels) was used; and third, the duration of the intervention was relatively short and consisted of only 8 weeks of intervention and 1 week of follow-up.

The future research development can be made in three directions: first, it is necessary to develop a broader sample, involving students of other geography and type of universities to generate more reliable external validity of studies; second, a longer intervention and follow-up can be chosen, with long-term follow-ups of 3 months and 6 months, to study the impact of systematic interventions; and third, multimodal research methods can be considered combining physiological indicators (heart rate, EEG) and behavioral indicators (duration of intervention, speed of feedback) to gain a more accurate assessment Moreover, the contact of the system with university mental health education models can be considered, including one linking the system with post-freshmen mental health screening in the interventions, which would create the whole chain of services of screening-intervention-referral.

## Conclusion

The adaptive language model-based mindfulness-based cognitive therapy (MBCT) system designed in this study follows the four-stage model of perception analysis and intervention feedback to dynamically and precisely generate and optimize intervention programs for the individual. Empirical studies indicate that this system can significantly reduce anxiety rates among college students, increase mindfulness, reduce negative automatic thoughts, and lead to greater satisfaction with and adherence of the intervention compared with conventional MBCT. Besides offering an efficient and convenient novel tool for anxiety interventions among college students, the study provides theoretical and real-world justification for the use of large language models in the mental health sector. As system functions continue to be optimized and the areas of application continue to grow, this will provide a significant input to the digitalization of the college mental health service systems.

## Data Availability

The raw data supporting the conclusions of this article will be made available by the authors, without undue reservation.

## References

[ref1] Brown T. B. Mann B. Ryder N. Subbiah M. Kaplan J. Dhariwal P. . (2020). Language models are few-shot learners. Adv. Neural Inf. Proces. Syst. 33, 1877–1901.

[ref2] Brown K. W. Ryan R. M. (2003). The benefits of being present: mindfulness and its role in psychological well-being. J. Pers. Soc. Psychol. 84, 822–848. doi: 10.1037/0022-3514.84.4.822, 12703651

[ref3] Carron-Arthur B. Ali K. Cunningham J. A. Griffiths K. M. (2015). From help-seekers to influential users: a systematic review of participation styles in online health communities. J. Med. Internet Res. 17:e271. doi: 10.2196/jmir.4705, 26627369 PMC4704975

[ref4] Chandrashekar P. (2018). Do mental health mobile apps work: evidence and recommendations for designing high-efficacy mental health mobile apps. mHealth 4, 1–10. doi: 10.21037/mhealth.2018.03.02, 29682510 PMC5897664

[ref5] Chiesa A. Serretti A. (2011). Mindfulness based cognitive therapy for psychiatric disorders: a systematic review and meta-analysis. Psychiatry Res. 187, 441–453. doi: 10.1016/j.psychres.2010.08.011, 20846726

[ref6] Firth J. Torous J. Nicholas J. Carney R. Pratap A. Rosenbaum S. . (2017). The efficacy of smartphone-based mental health interventions for depressive symptoms: a meta-analysis of randomized controlled trials. World Psychiatry 16, 287–298. doi: 10.1002/wps.20472, 28941113 PMC5608852

[ref7] Fulmer R. Joerin A. Gentile B. Lakerink L. Rauws M. (2018). Using psychological artificial intelligence (Tess) to relieve symptoms of depression and anxiety: randomized controlled trial. JMIR Mental Health 5:e64. doi: 10.2196/mental.9782, 30545815 PMC6315222

[ref8] Garrido S. Millington C. Cheers D. Boydell K. Schubert E. Meade T. . (2019). What works and what doesn’t work? A systematic review of digital mental health interventions for depression and anxiety in young people. Front. Psych. 10:759. doi: 10.3389/fpsyt.2019.00759, 31798468 PMC6865844

[ref9] Guntuku S. C. Schneider R. Pelullo A. Young J. Wong V. Ungar L. . (2019). Studying expressions of loneliness in individuals using twitter: an observational study. BMJ Open 9:e030355. doi: 10.1136/bmjopen-2019-030355, 31685502 PMC6830671

[ref10] Hofmann S. G. Sawyer A. T. Witt A. A. Oh D. (2010). The effect of mindfulness-based therapy on anxiety and depression: a meta-analytic review. J. Consult. Clin. Psychol. 78, 169–183. doi: 10.1037/a0018555, 20350028 PMC2848393

[ref11] Hoge E. A. Bui E. Marques L. Metcalf C. A. Morris L. K. Robinaugh D. J. . (2013). Randomized controlled trial of mindfulness meditation for generalized anxiety disorder: effects on anxiety and stress reactivity. J. Clin. Psychiatry 74, 786–792. doi: 10.4088/JCP.12m08083, 23541163 PMC3772979

[ref12] Hollon S. D. Kendall P. C. (1980). Cognitive self-statements in depression: development of an automatic thoughts questionnaire. Cogn. Ther. Res. 4, 383–395. doi: 10.1007/BF01178214

[ref13] Kabat-Zinn J. (2003). Mindfulness-based interventions in context: past, present, and future. Clin. Psychol. Sci. Pract. 10, 144–156. doi: 10.1093/clipsy.bpg016, 40388063

[ref14] Karyotaki E. Riper H. Twisk J. Hoogendoorn A. Kleiboer A. Mira A. . (2017). Efficacy of self-guided internet-based cognitive behavioral therapy in the treatment of depressive symptoms: a meta-analysis of individual participant data. JAMA Psychiat. 74, 351–359. doi: 10.1001/jamapsychiatry.2017.0044, 28241179

[ref15] Kessler R. C. Amminger G. P. Aguilar-Gaxiola S. Alonso J. Lee S. Ustun T. B. (2007). Age of onset of mental disorders: a review of recent literature. Curr. Opin. Psychiatry 20, 359–364. doi: 10.1097/YCO.0b013e32816ebc8c, 17551351 PMC1925038

[ref16] Khosrowabadi R. Quek C. Ang K. K. Wahab A. (2023). An adaptive AI-driven mindfulness intervention system for anxiety management: a randomized controlled trial. J. Med. Internet Res. 25:e40123.

[ref17] Lattie E. G. Adkins E. C. Winquist N. Stiles-Shields C. Wafford Q. E. Graham A. K. (2019). Digital mental health interventions for depression, anxiety, and enhancement of psychological well-being among college students: systematic review. J. Med. Internet Res. 21:e12869. doi: 10.2196/12869, 31333198 PMC6681642

[ref18] Levin M. E. Haeger J. A. Pierce B. G. Twohig M. P. (2017). Web-based acceptance and commitment therapy for mental health problems in college students: a randomized controlled trial. Behav. Modif. 41, 141–162. doi: 10.1177/0145445516659645, 27440189

[ref19] Li H. Zhang Y. Wang Q. (2024). Evaluating the efficacy of large language models in psychological assessment: a comparative study. Comput. Hum. Behav. 150:107998.

[ref20] Linardon J. Cuijpers P. Carlbring P. Messer M. Fuller-Tyszkiewicz M. (2019). The efficacy of app-supported smartphone interventions for mental health problems: a meta-analysis of randomized controlled trials. World Psychiatry 18, 325–336. doi: 10.1002/wps.20673, 31496095 PMC6732686

[ref21] Miner A. S. Haque A. Fries J. A. Fleming S. L. Wilfley D. E. Wilson G. T. . (2020). Assessing the accuracy of automatic speech recognition for psychotherapy. NPJ Digit. Med. 3, 1–8. doi: 10.1038/s41746-020-0285-8, 32550644 PMC7270106

[ref22] Radford A. Narasimhan K. Salimans T. Sutskever I. (2018). Improving Language Understanding by Generative Pre-Training. San Francisco, CA, USA: OpenAI.

[ref23] Richards D. Richardson T. (2012). Computer-based psychological treatments for depression: a systematic review and meta-analysis. Clin. Psychol. Rev. 32, 329–342. doi: 10.1016/j.cpr.2012.02.004, 22466510

[ref24] Segal Z. V. Williams J. M. G. Teasdale J. D. (2002). Mindfulness-Based Cognitive Therapy for Depression: A New Approach to Preventing Relapse. New York, NY: Guilford Press.

[ref25] Spielberger C. D. Gorsuch R. L. Lushene R. Vagg P. R. Jacobs G. A. (1983). Manual for the State-Trait Anxiety Inventory (STAI). Palo Alto, CA: Consulting Psychologists Press.

[ref26] Torous J. Nicholas J. Larsen M. E. Firth J. Christensen H. (2018). Clinical review of user engagement with mental health smartphone apps: evidence, theory and improvements. Evid. Based Ment. Health 21, 116–119. doi: 10.1136/eb-2018-102891, 29871870 PMC10270395

[ref27] Twomey C. O’Reilly G. Byrne M. (2015). Effectiveness of cognitive behavioural therapy for anxiety and depression in primary care: a meta-analysis. Fam. Pract. 32, 3–15. doi: 10.1093/fampra/cmu060, 25248976

[ref28] Wang K. Varma D. S. Prosperi M. (2018). A systematic review of the effectiveness of mobile apps for monitoring and management of mental health symptoms or disorders. J. Psychiatr. Res. 107, 73–78. doi: 10.1016/j.jpsychires.2018.10.006, 30347316

[ref29] Zhang Y. Li W. Wang J. (2022). The effect of mindfulness-based cognitive therapy on anxiety and depression in college students: a meta-analysis of randomized controlled trials. J. Affect. Disord. 318, 1–6. doi: 10.1016/j.jad.2022.08.130, 36057283

[ref30] Zung W. W. K. (1971). A rating instrument for anxiety disorders. Psychosomatics 12, 371–379. doi: 10.1016/S0033-3182(71)71479-0, 5172928

